# Genomic Sequencing of Japanese Plum (*Prunus salicina* Lindl.) Mutants Provides a New Model for Rosaceae Fruit Ripening Studies

**DOI:** 10.3389/fpls.2018.00021

**Published:** 2018-02-19

**Authors:** Angel Fernandez i Marti, Christopher A. Saski, George A. Manganaris, Ksenija Gasic, Carlos H. Crisosto

**Affiliations:** ^1^Department of Plant Sciences, University of California, Davis, Davis, CA, United States; ^2^Genomics and Computational Biology Laboratory, Biosystems Research Complex, Clemson, SC, United States; ^3^Department of Agricultural Sciences, Biotechnology & Food Science, Cyprus University of Technology, Lemesos, Cyprus; ^4^Department of Plant and Environmental Sciences, Clemson University, Clemson, SC, United States

**Keywords:** SNPs/INDELS, copy number variation, PE Illumina libraries, climacteric, suppressed-climacteric, non-climacteric, ripening, molecular evolution

## Abstract

It has recently been described that the Japanese plum “Santa Rosa” bud sport series contains variations in ripening pattern: climacteric, suppressed-climacteric and non-climacteric types. This provides an interesting model to study the role of ethylene and other key mechanisms governing fruit ripening, softening and senescence. The aim of the current study was to investigate such differences at the genomic level, using this series of plum bud sports, with special reference to genes involved in ethylene biosynthesis, signal transduction, and sugar metabolism. Genomic DNA, isolated from leaf samples of six Japanese plum cultivars (“Santa Rosa”, “July Santa Rosa”, “Late Santa Rosa”, “Sweet Miriam”, “Roysum”, and “Casselman”), was used to construct paired-end standard Illumina libraries. Sequences were aligned to the *Prunus persica* genome, and genomic variations (SNPs, INDELS, and CNV's) were investigated. Results determined 12 potential candidate genes with significant copy number variation (CNV), being associated with ethylene perception and signal transduction components. Additionally, the Maximum Likelihood (ML) phylogenetic tree showed two sorbitol dehydrogenase genes grouping into a distinct clade, indicating that this natural group is well-defined and presents high sequence identity among its members. In contrast, the ethylene group, which includes ACO1, ACS1, ACS4, ACS5, CTR1, ERF1, ERF3, and ethylene-receptor genes, was widely distributed and clustered into 10 different groups. Thus, ACS, ERF, and sorbitol dehydrogenase proteins potentially share a common ancestor for different plant genomes, while the expansion rate may be related to ancestral expansion rather than species-specific events. Based on the distribution of the clades, we suggest that gene function diversification for the ripening pathway occurred prior to family extension. We herein report all the frameshift mutations in genes involved in sugar transport and ethylene biosynthesis detected as well as the gene CNV implicated in ripening differences.

## Introduction

Ripening is a highly synchronized and genetically regulated stage of fruit development that precedes senescence. Typical physical and chemical changes during ripening include (i) degradation of cell walls (softening), (ii) color alteration through changes in chlorophyll, carotenoid, and flavonoid accrual, and (iii) modifications of sugar-acid metabolism and synthesis of aromatic volatiles that enhance flavor (Giovannoni, 2004). There is a consensus that synthesis and perception of ethylene are imperative for ripening-related changes in climacteric type fruits, including softening and flavor development. The biosynthetic pathway for ethylene is relatively simple and involves two enzymes: ACC synthase (ACS), which converts S-adenosylmethionine (SAM) to 1-aminocyclopropane-1-carboxylate (ACC), and ACC oxidase (ACO), which converts ACC to ethylene. Even though the pathway does not entail many steps, these few steps are highly regulated. Several genes are involved in ACS and ACO transcription and the conversion of SAM to ACC is the rate-limiting step (Klee and Giovannoni, 2011); thus, levels of ACS transcription correlate directly with ethylene production. During initiation of ripening in climacteric-type fruits, genes that express transcription factors (TFs) that promote ACS synthesis are expressed. These TFs are then transcribed and translated into proteins that bind with the promoter region of the DNA, responsible for expressing ACS synthesis. For ethylene-induced ripening responses to occur, the presence of the phytohormone must also be perceived and this signal must be transduced to other parts of the fruit. Genetic analysis of *Arabidopsis* and tomato concluded that ethylene receptors act as negative regulators of the ethylene response pathway (Hamilton et al., 1990; Tieman et al., 2000). Thus, delaying endogenous ethylene production or its perception by receptors should delay ripening/softening changes, allowing fruit to stay longer on the tree.

Japanese plum (*Prunus salicina*) is considering a climacteric type fruit, depicting a typical burst on ethylene synthesis at the onset of ripening (Manganaris et al., 2008). Most commercial Japanese plums bear climacteric fruit that exhibit autocatalytic production of endogenous ethylene, characterized by color changes, fast ripening/softening that may lead to fruit drop when harvesting is delayed. Interestingly, a ripening type that is intermediate between climacteric and non-climacteric has been described in plums and named suppressed-climacteric type (Abdi et al., 1997). Such fruit type is characterized by low levels of ethylene evolution and respiration rate, but when exposed to exogenous ethylene, suppressed-climacteric renew typical climacteric ripening (Abdi et al., 1997). Comprehensive studies dealt with genes involved in ethylene biosynthesis, perception and signal transduction, and responsive transcription in a suppressed-climacteric (“Shiro”) and a climacteric type cultivar (“Early Golden”; El-Sharkawy et al., 2007, 2008, 2009). Four ACS genes (*Ps-ACS1, Ps-ACS3, Ps-ACS4*, and *Ps-ACS5*), three ethylene perception and signal transduction components (*Ps-ETR1, Ps-ERS1*, and *Ps-CTR1*) and several ethylene-responsive transcription factors (*Ps-ERF1a, Ps-ERF1b, Ps-ERF2a, Ps-ERF2b, Ps-ERF3a, Ps-ERF3b*, and *Ps-ERF12*) were classified among these distinct groups.

A non-climacteric type plum cultivar, Sweet Miriam, has been recently reported by our group (Minas et al., 2015), belonging to a series of commercial plum cultivars that are bud sports of “Santa Rosa” (Okie and Ramming, 1999). Genetic analysis using 10 simple sequence repeat (SSR) markers produced identical DNA profiles for the climacteric cultivars Santa Rosa and July Santa Rosa, the suppressed-climacteric cultivars “Late Santa Rosa,” “Casselman” and “Roysum,” and the novel non-climacteric cultivar Sweet Miriam, suggesting that these cultivars are bud-sports of “Santa Rosa.” The SSR markers used were able to distinguish closely related genotypes, but could not discriminate bud sport mutations (Minas et al., 2015). We believe that genetic polymorphisms in genes related to ethylene are the cause of the three ripening phenotypes observed in this cluster of plum bud sports.

The ripening behavior of this “Santa Rosa” series was investigated in the absence (air) or in the presence of ethylene or propylene (an ethylene analog) following treatment or not with 1-methylcyclopropene (1-MCP, an ethylene action inhibitor; Minas et al., 2015). Contrary to climacteric plum fruits, that of the slow-softening suppressed-climacteric cultivars “Late Santa Rosa,” “Casselman,” and “Roysum” produced detectable amounts of ethylene, while the novel non-climacteric “Sweet Miriam” produced no ethylene and softened extremely slowly, even after propylene exposure (Minas et al., 2015). In addition, sugar catabolism seems to be greatly affected, with fully ripe, non-climacteric fruit accumulating 2.5-fold higher amounts of the sugar alcohol sorbitol than climacteric fruit (Kim et al., 2015).

Most of the research in molecular regulation of fruit ripening has been done in tomato, where mutations blocking the transition to ripe fruits have facilitated understanding of the role of ethylene and its associated molecular networks in the control of ripening (Osorio et al., 2013). Suitable tree fruit mutations to study ripening and postharvest characteristics have been scarce. Stony hard (SH) peach phenotype is mutant characterized by lack of ethylene production that has been included in many breeding programs as a genetic source for improving quality traits of peaches (Haji et al., 2001). The SH phenotype has been researched extensively for the promise of developing firmer peaches with tree-ripe flavor and longer storage (Tatsuki et al., 2013; Pan et al., 2015). Most research on the stony hard trait in peach has been conducted using segregating progeny from crosses between climacteric and stony hard cultivars, which exposed the need for peach mutants to develop new knowledge on the control of ripening and ethylene. Japanese plum, a related yet phenotypically distinct species, appears as an interesting model to study fruit ripening and further dissect the role of ethylene at the perception and signal transduction levels.

Our approach-working hypothesis is that Rosaceae fruit quality and flavor could be improved if the fruits remain on the tree longer, allowing accumulation of desired sugars, antioxidants, and bioactive compounds without excessive softening. Thus, if the climacteric response of the ethylene-induced ripening process can be controlled while fruits are on the tree, ripening/softening can be optimized to increase consumer fruit quality. We hypothesize that polymorphisms in the coding regions of genes related to ethylene synthesis, perception, signal transduction and transcription are the genetic cause of the three ripening phenotypes observed in the six sport cultivars of Japanese plums.

Therefore, the aim of the current study is to use “Santa Rosa” mutant series to understand copy number variations (CNVs) in key softening-related genes that govern ethylene perception and signal transduction during plum fruit ripening in three different ripening patterns; climacteric, suppressed-climacteric and non-climacteric types. To this end, Whole-genome shotgun (WGS) sequencing has been carried out in 6 plum cultivars. Finally, a phylogenetic approach was undertaken in order to investigate molecular adaptation of the sorbitol and ethylene genes across different plant genomes.

## Materials and methods

### Plant material, genomic DNA extraction and sequencing

Leaf samples were obtained from six cultivars of Japanese plum (*P. salicina* Lindl): “Santa Rosa” (SR), “July Santa Rosa (JSR),” “Late Santa Rosa (LSR),” “Casselman,” “Roysum,” and “Sweet Miriam (SM),” all growing at the UC Pomology farm at Davis, CA. Full agronomical, organoleptic, nutritional, postharvest, and physiological characterization of the ripening behavior and the softening regulation of the six different plum types studied in this work have previously been described (Minas et al., 2015).

Genomic DNA of the six cultivars was isolated from leaves using standard Cetyl Trimethylammonium Bromide (CTAB) methods (Lodhi et al., 1994). Five micro gramstotal gDNA was sheared to a fragment size of ~600 bp with a Covaris ultrasonicator (Aubakirova et al., 2012) and Illumina-ready sequencing libraries were prepared using the Illumina TruSeq-DNA library preparation kit, following the manufacturer's recommended procedures. WGS sequences were collected on an Illumina HiSeq2500 using a 2 × 125 bp paired-end module.

### Bioinformatic analysis

After sequencing, raw sequence reads were subject to quality analysis with FastQC software (http://www.bioinformatics.babraham.ac.uk/projects/fastqc/), and pre-processed to remove low-quality bases and adapter sequences with the Trimmomatic tool (Bolger et al., 2014). Pre-processed sequence reads were aligned to the *Prunus persica* (Verde et al., 2017) reference genome assembly v. 2.0 using the Bowtie2 short read aligner tool (Langmead and Salzberg, 2012). Gene CNV was performed with a sliding window of 100 bp and the CNVnator prediction algorithm (Abyzov et al., 2011). CNVnator output was converted to tabular format with in-house scripts and combined with the peach gene annotation files for analysis of genes. Coding variations (SNPs and INDELS), both relative to peach and among the plum samples, were determined with UnifiedGenotyper, a genotyping walker in the Genome Analysis Tool Kit (DePristo et al., 2011) with output in vcf format. Variants were filtered for depth (DP5) and mapping quality (MQ30), using in-house scripts. Functional annotations of mutations were determined with the SNPeff and SNPsift software tools (Cingolani et al., 2012). Variant sites were removed where all six plum varieties shared the same genotype, creating a final variant file output that contains sites where at least one variety differs from the other five using in-house scripts (Supplementary Table 3, 4). Relative SNP densities were determined using a 100 kb window and plotted using the Circos plotting tool (Krzywinski et al., 2014).

### Evolutionary phylogenetic analysis and positive selection tests

Predicted proteome sequences from Citrus clementine, *Solanum lycopersicum, Fragaria vesca, Malus domestica, Arabidopsis thaliana, Vitis vinifera, P. persica, Pinus taeda*, and *Carica papaya* were selected and downloaded from phytozome (https://phytozome.jgi.doe.gov/pz/portal.html). The interProScan 4-package software (https://www.ebi.ac.uk/interpro/) was used to identify the proteins in each proteome dataset. Local databases for each included plant species were developed to allow us to extract and interpret the large amount of data obtained in this study. Out of all loci identified, nine for ethylene and two for sorbitol responses were clearly differentiated among all genotypes used in this study. These 11 sequences were used as a reference sequences to explore orthologs in the nine plant species mentioned above. Sequences of the best BLAST hits for each gene/species were aligned using ClustalW integrated within the program Geneious. Best hits were considered as the first ones within an interval of 99 “% max similarity” and 30 for “% min similarity.” The aligned sequences were visualized and manually refined using Jalview software (www.jalview.org). Phylogenetic analyses were performed using the maximum likelihood method through the customizable version of RAxML 8.0 (Stamatakis, 2014). Paralogous gene pairs were determined by protein phylogeny and used as reference for a multiple alignment of DNA coding sequences using ClustalW. KaKs_Calculator software (Zhang et al., 2006) was used to determine the ratio of non-synonymous to synonymous mutations (dN/dS ratio or), representing the selective selection pressures: neutral (ω = 1), purifying (ω < 1) or positive (ω > 1).

### Representation of chromosomal map

The SNP (single nucleotide polymorphism) and INDELS (insertion/deletion) markers identified in this study were further filtered for chromosomal map construction. SNP and INDELS, different between “Santa Rosa” and the sports, were placed physically in the peach reference genome. The location (in bp) of each marker was retrieved from the peach genome and anchored to the map. Genes associated with ethylene and sorbitol were designated in purple. The physical map was drawn using MapChart 2.3 software (Voorrips, 2002).

## Results

### Resequencing of the varieties and variant discovery

Whole-genome shotgun sequencing data was collected for each of the six plum cultivars to a ~38 X depth, relative to the reference *P. persica* genome assembly (Supplementary Table 1). A total of 174,570 variant sites were identified after filtering for depth, allele frequency, mapping quality, coding region, and the requirement for at least one cultivar to contain a discriminatory genotype from the others. “Casselman” had the most SNP variants (5,718), followed by “July Santa Rosa” (5,496), and “Late Santa had the fewest at 3,259 (Supplementary Table 2 and Supplementary Figure 1). Insertion/Deletion (INDEL) variants were less frequently present, but the abundances followed a similar pattern, with “Casselman” containing the most INDELs (2,855), followed by “July Santa Rosa” (2,714), and “Late Santa Rosa” having the fewest (1,905) (Supplementary Table 2). A neighbor-joining analysis using the complete set of SNP profiles among the cultivars demonstrated that “Casselman” and “July Santa Rosa” are the most closely related forming a sub-group from “Roysum” and “Santa Rosa”; which appear to have diverged from a split between “Late Santa Rosa” and “Sweet Miriam” (Supplementary Figure 2). Variant effects differed extensively among cultivars (Supplementary Table 2). Missense variation, which are point mutations that alter the amino acid code, were the predominant variation result, with the plum cultivars containing between 1,000 and 2,000. Synonymous SNP variants ranged from 688 to 1,221, while we detected only up to 143 as a result of INDEL variation (Supplementary Table 2). Variation in the untranslated regions (5′ and 3′ UTRs) was much greater in the 3′ regions, which implies possible alterations in the regulatory features of these genes (Barrett et al., 2012). Several high-impact mutations, such as stop codon gains and losses, start losses, and splice site modifications, were also detected (Supplementary Table 2) and are described in detail in the sections below.

### Prioritizing variant effects and copy number variation

Candidate mutations and genes were prioritized, using an evidence-based approach that takes into account the variant effect. After discarding the non-relevant SNPs with effects such as conservative in-frame insertion, intron variant, and intergenic region, 280 SNPs and 116 INDELS were selected as candidate variants that could serve as possible casual mutations for a non-synonymous variant, stop gained, missense variant, downstream gene variation, 5′prime UTR premature start codon gain variant, disruptive in-frame deletion or frameshift variant (Tables 1, 2 and Supplementary Tables 6–9).

**Table 1:** Description of gene function and number of loci found to share the same function after filtering the putative sequence variants (SNP and INDELs) distributed the examined cultivars “Santa Rosa”, “July Santa Rosa”, “Late Santa Rosa”, “Casselman”, “Roysum”, and “Sweet Miriam”.

**Gene Function**	**N loci**	**Gene Function**	**N loci**
Sugar/inositol transporter	16	Carbohydrate/puine kinase, PfkB, conserved site	1
Protein kinase domain	13	Cation efflux protein	1
Cellulose synthase	11	CCAAT-binding factor	1
Glycoside hydrolase family 38, N-terminal domain	11	CS domain	1
Glycosyltransferase, DXD sugar-binding motif	3	Cyclic nucleotide-binding domain	1
NAD-dependent epimerase/dehydratase, N-terminal domain	3	D-galactoside/L-rhamnose binding SUEL lectin domain	1
Peptidase S8/S53 domain	3	Development/cell death domain	1
Protein phosphatase 2C (PP2C)-like domain	3	Flavoprotein	1
Ribosomal protein L1, 2-layer alpha/beta-sandwich	3	Forkhead-associated (FHA) domain	1
Small GTPase superfamily	3	Fumarate lyase family	1
WD40 repeat	3	GAF domain	1
Zinc finger, C2H2	3	GDP-fucose protein O-fucosyltransferase	1
AAA+ ATPase domain	2	GH3 auxin-responsive promoter	1
AP2/ERF domain	2	Heavy metal-associated domain, HMA	1
Armadillo-like helical	2	Helicase, C-terminal	1
Bacterial transferase hexapeptide repeat	2	Histidine phosphatase superfamily, clade-1	1
Carbohydrate kinase PfkB	2	Hs1pro-1, C-terminal	1
Decaprenyl diphosphate synthase-like	2	Inositol polyphosphate-related phosphatase	1
Pentatricopeptide repeat	2	Isocitrate and isopropylmalate dehydrogenases family	1
Peptidyl-prolyl cis-trans isomerase, FKBP-type, domain	2	Isopenicillin N synthase	1
Phox/Bem1p	2	JmjC domain	1
Protein phosphatase 2C	2	LysM domain	1
Reticulon	2	Major facilitator superfamily	1
Ribonuclease H domain	2	Methylenetetrahydrofolate reductase	1
SANT/Myb domain	2	Myc-type, basic helix-loop-helix (bHLH) domain	1
SET domain	2	Pathogenic type III effector avirulence factor Avr cleavage site	1
SOUL haem-binding protein	2	Phosphate permease	1
Sucrose synthase	2	Plant disease resistance response protein	1
Transcription factor, MADS-box	2	PQ-loop repeat	1
Tubulin	2	Pre-rRNA-processing protein TSR2	1
Protein family UPF0497, trans-membrane plant	2	Reversibly glycosylated polypeptide family	1
UTP–glucose-1-phosphate uridylyltransferase family	2	Rhodanese-like domain	1
Alcohol dehydrogenase superfamily, zinc-type	2	Ribokinase	1
Phosphate transporter	2	RNA recognition motif domain	1
Proteasome alpha-subunit, N-terminal domain	2	RNA-binding, CRM domain	1
Sugar phosphate transporter	2	Sec39 domain	1
6-phosphogluconolactonase, DevB-type	1	Short-chain dehydrogenase/reductase SDR	1
Actin family	1	Signal transduction response regulator, receiver domain	1
Acyl-CoA-binding protein, ACBP	1	Sin3 associated polypeptide p18	1
Acylneuraminate cytidylyltransferase	1	Superoxide dismutase, copper/zinc binding domain	1
Adenosine kinase	1	SWAP/Surp	1
Adenylate kinase/UMP-CMP kinase	1	SWEET sugar transporter, plants	1
Alpha crystallin/Hsp20 domain	1	TATA element modulatory factor 1 TATA binding	1
Aminotransferases, class-I, pyridoxal-phosphate-binding site	1	Tetrahydrofolate dehydrogenase/cyclohydrolase	1
AMP-dependent synthetase/ligase	1	Tetratricopeptide-like helical domain	1
Ankyrin repeat	1	Thioredoxin	1
Armadillo	1	Transcription factor GRAS	1
ATPase, V1 complex, subunit F, eukaryotic	1	Transcription factor, SBP-box	1
AUX/IAA protein	1	Translation elongation factor EFG, V domain	1
Basic-leucine zipper domain	1	Trehalose-phosphatase	1
BRCT domain	1	UAA transporter	1
cAMP response element binding (CREB) protein	1	Yippee/Mis18	1
Unknown	89		

**Table 2:** Description of gene effect and number of potential variants per effect associated with the biosynthetic pathway of fruit ripening in plum.

**Gene effect**	**SNP**	**INDELS**
synonymous_variant	30	0
stop_gained	3	0
missense_variant	26	0
downstream_gene_variant	217	107
5_prime_UTR_premature_start_codon_gain_variant	4	0
disruptive_inframe_deletion	0	2
downstream_gene_variant	0	0
frameshift_variant	0	6
stop_gained & disruptive_inframe_insertion	0	1
Total	280	116

Among the variant sites, 10 were candidate genes associated with different ethylene and sorbitol responses between the two main phenotypes (climacteric vs. non-climacteric). These potentially affected regulation of ACC synthase (ACS), ethylene receptors (ETR), constitutive triple response (CTR) genes and sorbitol dehydrogenase (SDH) genes (Table 3).

**Table 3:** Description (target gene, PFAM, linkage group and number of copies) of the 11 ethylene and two sorbitol candidate genes across the examined cultivars [cvs. “Santa Rosa” (SR), “July Santa Rosa” (JSR), “Late Santa Rosa” (LSR), “Casselman,” “Roysum,” and “Sweet Miriam” (SM)].

**Peach Gene**	**Plum targeted genes**	**Plum Gene Orth. Annot**	**PFAM**	**LG**	**Old Peach name**	**Climacteric**		**Climacteric–4 days slow**	**Slow 6 days**	**Non-climacteric**	**Suppressed**
						**Copies in JSR**	**Copies in SR**	**Copies in LSR**	**Copies in Casselman**	**Copies in SM**	**Copies in Roysum**
Prupe.3G209900	Ps-ACO1_(KM030036.1)	AC01	2OG-Fe(II) oxygenase superfamily	Pp03	ppa008791m	6.6	3.5	6.6	0.5	3.6	4.0
Prupe.6G214400	Ps-ACS1_(EU034649)	ACS1	Aminotransferase class I and II	Pp06	ppa004987m	0.7	1.1	0.3	0.8	0.3	1.3
Prupe.2G176900	Ps-ACS4_(EU034653)	ACS4	Aminotransferase class I and II	Pp02	ppa004774m	0.5	4.0	0.2	0.5	0.0	4.0
Prupe.5G106200	Ps-ACS5_(EU034654)	ACS5	Aminotransferase class I and II	Pp05	ppa016458m	0.9	4.1	3.9	0.0	3.9	3.9
Prupe.7G117700	Ps-CTR1_(EF585298)	CTR1	Ethylene-responsive protein kinase Le-CTR1	Pp07	ppa001532m	0.0	3.2	4.1	0.0	4.0	3.6
Prupe.4G051200	Ps-ERF1_(EF607278)	ERF	AP2 domain	Pp04	ppa012385m	8.0	3.7	3.5	4.1	3.5	3.6
Prupe.5G061800	Ps-ERF1a_(FJ026009)	ERF1a	AP2 domain	Pp05	ppa009707m	3.6	4.2	4.1	3.9	4.3	4.2
Prupe.2G272300	Ps-ERF1b_(FJ026008)	ERF1b	AP2 domain	Pp02	ppa010186m	0.4	0.7	0.4	3.5	4.1	1.0
Prupe.1G556000	Ps-ETR1	ethy_receptor	GAF domain	Pp01	ppa001917m	0.8	4.0	1.2	3.5	1.0	5.5
Prupe.3G209100	Ps-ERF3	ERF3	AP2 domain	Pp03	ppa010804m	0.3	0.5	0.3	4.4	4.4	0.6
Prupe.2G288800	Ps-Sorbitol1	Sorbitol dehydrogenase	Alcohol dehydrogenase GroES-like domain	Pp02	ppa007458m	0.3	3.8	0.3	0.2	0.6	3.6
Prupe.4G240300	Ps-Sorbitol2	Sorbitol dehydrogenase	Alcohol dehydrogenase GroES-like domain	Pp04	ppa007327m	1.0	0.5	0.0	0.0	4.2	1.1

**Figure 1:**
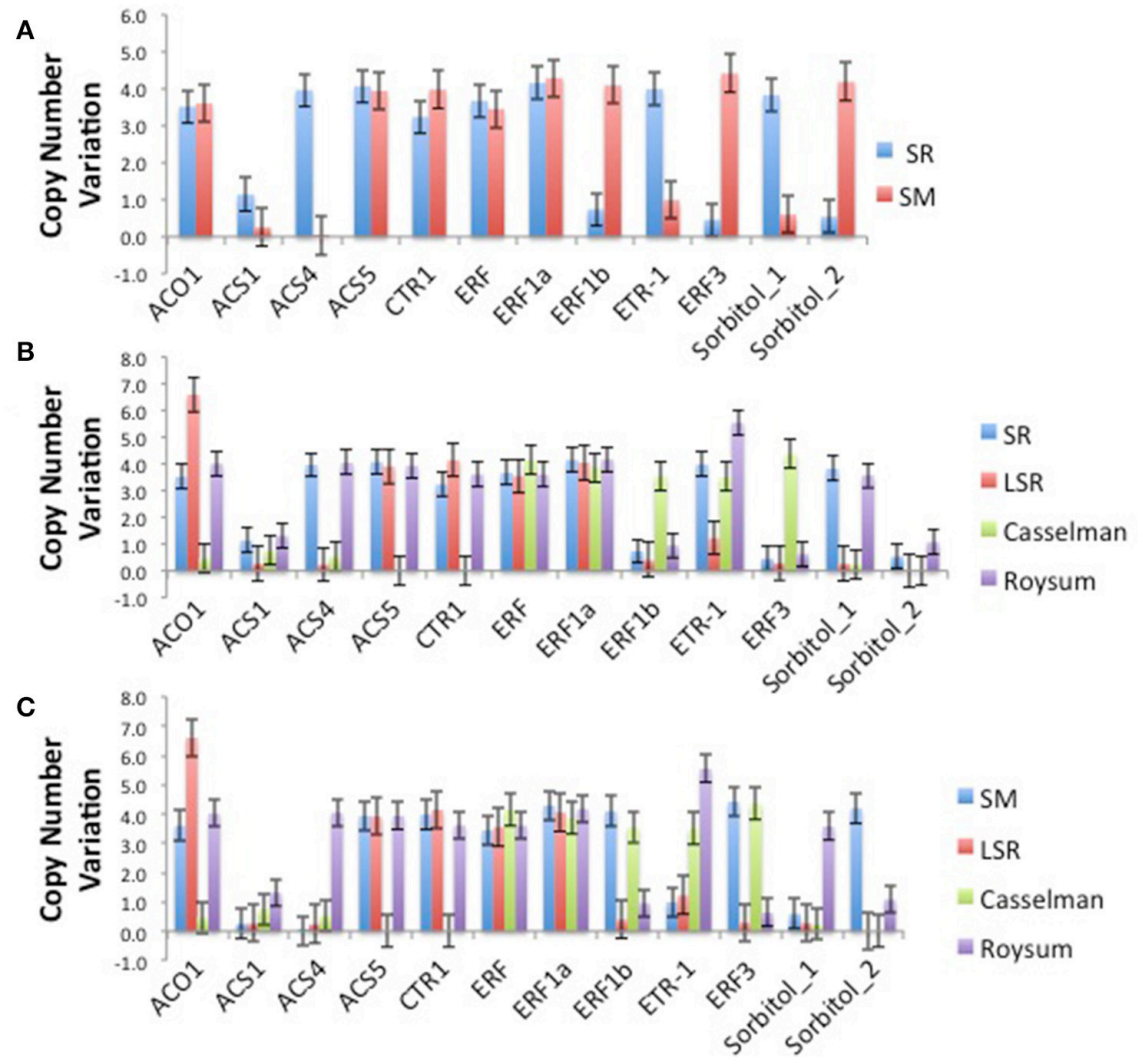
**(A)** Comparison of the climacteric “Santa Rosa” and non-climacteric “Sweet Marian” plum cultivars, based on their number of copies for each particular candidate genes related to ethylene biosynthesis and perception and sorbitol biosynthesis. **(B)** Comparison of the two main cultivars, the climacteric “Santa Rosa” and three suppressed-climacteric type cultivars (“Late Santa Rosa,” “Casselman,” “Roysun”) based on their number of copies for each particular candidate genes related to ethylene biosynthesis and perception and sorbitol biosynthesis. **(C)** Comparison of three suppressed-climacteric type cultivars (“Late Santa,” “Casselman,” “Roysun”) and nonclimacteric type of “Sweet Marian” based on their number of copies for each particular candidate genes related to ethylene biosynthesis and perception and sorbitol biosynthesis.

The climacteric cultivars SR and LSR had a high copy number of the ACO1 gene of 6.6 and 6.6 respectively, with non-climacteric SM (3.6) and supressed-climacteric “Roysum” (4.0) showing similar results (Figure 1). However, the suppressed-climacteric “Casselman” had only 0.5 copies of the same gene. Among the genes associated with aminotransferase class I and II (ACS1, ACS4, and ACS5), SR had in general more copies (1.1, 4.0, and 4.1 respectively) than its mutant LSR (Figure 1). Interestingly, the non-climateric “Sweet Miriam” exhibited 0.3, 0.0, and 3.9 copy numbers for the same genes, respectively (Figure 1). This difference, especially for ACS1 and ACS4 genes, represents a comprehensive mutation profile that may explain the different ripening patterns and a very low effect of these genes in SM. Significant differences were also found in the same genes in the suppressed cultivar “Casselman” (0.8, 0.5 and 0), and the other two climacteric cultivars, “July Santa Rosa” (0.7, 0.5, and 0.9) and “Late Santa Rosa” (0.3, 0.2, and 3.9; Figure 1). Similarly, significant differences in copy number of ERF1b and ERF3 were also observed: where climacteric SR had very few copies (0.7 and 0.5) compared to non-climacteric SM (4.1 and 4.4). The copy numbers in “July Santa Rosa” (0.4 and 0.3) and “Late Santa Rosa” (0.4 and 0.3) were also very low, as in SR. However, the suppressed “Casselman” was similar to SM, with values of 3.5 and 4.4, respectively.

Intriguingly, the ethylene receptor gene ETR1 CNV observed in climacteric SR (4.0) was very high compared to SM (1.0). The other two climacteric varieties, JSR with 0.8 and LSR with 1.2, had a low copy number similar to SM. On the other hand, the two candidate sorbitol genes (Ps-Sorbitol1 and Ps-Sorbitol2) presented different patterns. Ps-Sorbitol1 had a higher CNV in SR than in SM (3.8 and 0.6, respectively), with low values also observed in JSR (0.3), LSR (0.3), and “Casselman” (0.2). Ps-sorbitol2 had opposite results; SM had more copies than the climacteric SR (4.2 and 0.5, respectively). In the other examined cultivars, the climacteric JSR and LSR had low values of 1 and 0, respectively, and this pattern was also seen in supressed-climacteric “Casselman” and “Roysum,” with values of 0 and 1, respectively (Figure 1 and Supplementary Table 5).

### Candidate gene analyses

To search for a duplication mechanism for the ethylene and sorbitol genes, we examined their physical genomic location using the peach genome, Peach v2.0, as a reference and constructed a physical map with the distribution of the homogenous selected markers (SNP and INDELS) along the eight scaffolds.

Scaffold 1 had the most markers at 9,313 SNPs and 1,709 INDELS. Scaffold 2 had 3,836 SNPs and 592 INDELS; scaffold 3, 3,963 SNPs and 726 INDELS; scaffold 4, 3,764 SNPs and 606 INDELS; scaffold 5, 3,257 SNPs and 533 INDELS; and scaffold 6, 4,292 SNPs and 672 INDELS. Scaffold 7 was by far the shortest and had only 2,070 SNPs and 357 INDELS. Finally, scaffold 8 had the second longest group at 4,932 SNPs and 932 INDELS (Supplementary Figure 1).

**Figure 2:**
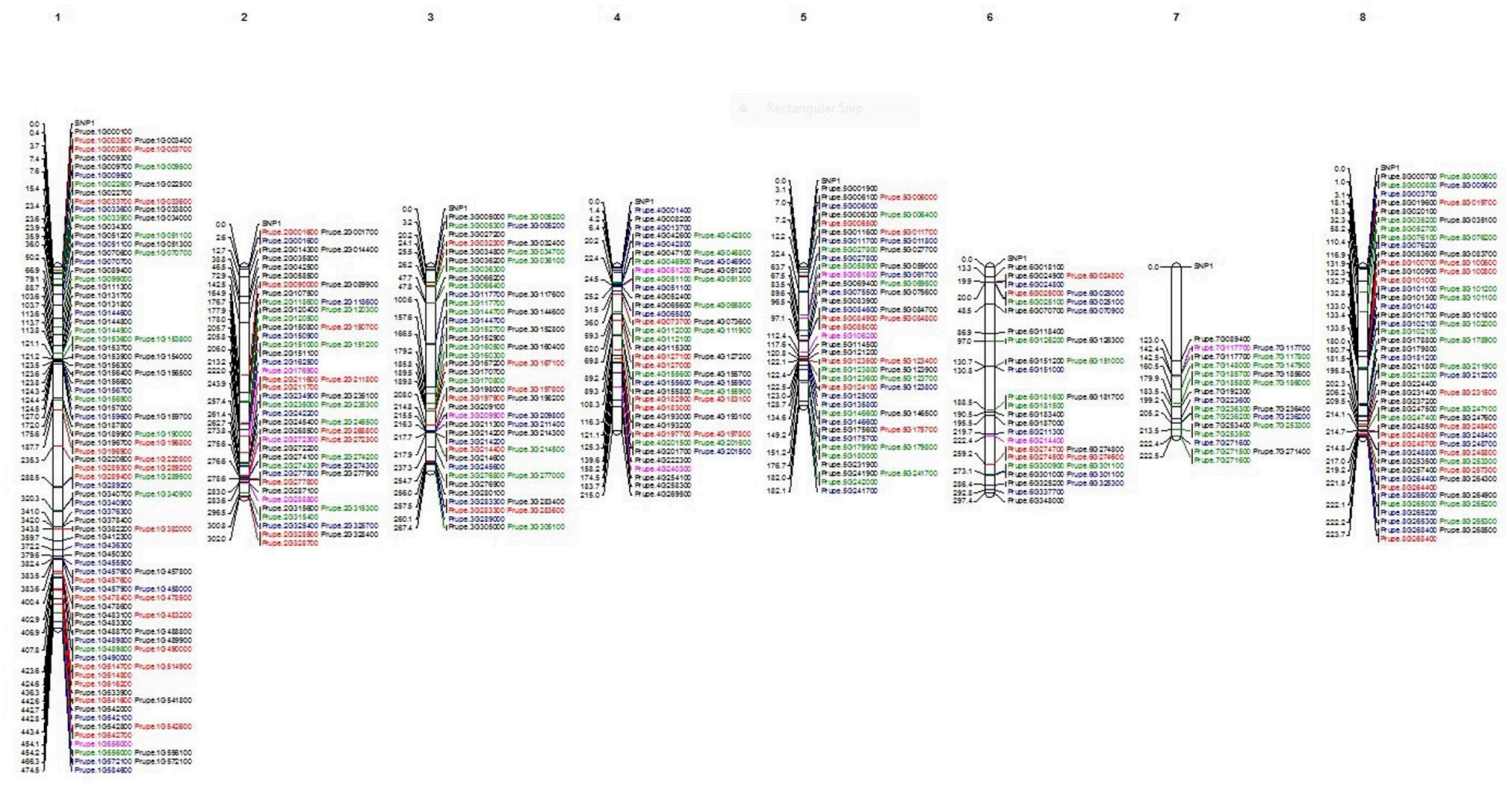
Physical map of Japanese plum using the filtered SNPs, INDELS and the 12 candidate genes highly associated to ethylene and sorbitol response. In black all the SNP found to be different between Santa Rosa and the rest of cultivars. In blue are shown the SNP where Santa Rosa are different to Sweet Mariam. In green all the SNP that confer differences between the climacteric and non-climateric cultivars. In red are shown all the INDELS found to be different between Santa Rosa and Sweet Mariam. In purple, are show the 12 candidate genes for ethylene and sorbitol response.

Out of the initial 35,431 SNPs and 16,740 INDELS, a group of 280 SNPs and 116 INDELS was used to construct a map (Figure 2). These markers were selected based on their possible influence on variation and high impact on the function of the proteins. All 280 SNPs used in the map are different between the climacteric cultivar SR and its bud sports (in black). Among them, 108 SNPs (in green) were different between the two most-contrasting cultivars in this study, climacteric SR and its mutant, the non-climateric SM (green). The 205 SNPs (red) were different between the group of climacteric cultivars (SR, LSR, JSR, “Roysum,” and “Casselman”) and the non-climacteric cultivar SM. A total of 116 INDELs exhibited differences between SR and the mutants (shown in blue). Finally, the two candidate genes that may be responsible for differential expression during ripening of ethylene and sorbitol are marked in purple. As expected, the coverage per scaffold was longest in chromosome 1. Group 1 (G1) representing scaffold 1 was the longest at 108 markers. This group contained 24 SNPs that differed between SR and SM and the candidate gene Prupe.1G556000 (old nomenclature: ppa001917m), a known ethylene receptor with a GAF domain. Group 2 (G2) was comprised of 56 SNPs and INDELS, of all categories, and three candidate genes. Two are in the ethylene pathway: Prupe.2G176900, an ACS4 gene based on *Pfam* and aminotransferase class I and II (old nomenclature: ppa004774m) and an ERF gene with an AP2 domain, Prupe.2G272300 (old nomenclature: ppa010186m). The third gene is one of the two sorbitol genes, Prupe.2G288000, which has an alcohol dehydrogenase GroEs-like domain (old nomenclature: ppa007458m). Scaffold 3 (G3) was also well represented with 47 SNPs and nine INDELS that differed between SR and SM and between climacteric and non-climateric phenotypes. Also in this group was an ethylene candidate gene associated with the 2OG-Fe(II) oxygenase superfamily ACO1 (Prupe.3G209900; old nomenclature: ppa008791m). G4 comprised 40 SNPS, 10 INDELS, and another candidate gene for ethylene, an ERF with an AP2 domain at the upper part, Prupe.4G051200 (old nomenclature: ppa012385m). The second sorbitol dehydrogenase gene was also found at the end of G4 (Prupe.4G240300; old nomenclature: ppa007327m). Two other ethylene genes, ACS5 (Prupe.5G106200; old nomenclature: ppa016458m) of the aminotransferase classes I and II and another ethylene receptor gene with an AP2 domain (Prupe.5G061800; old nomenclature: ppa009707m) were found on the scaffold five (G5). This group was represented by 40 SNPs of all categories and 15 INDELS. Scaffold 6 (G6) was the second-shortest group at only 25 SNPs and nine INDELS, but its upper part hosted ethylene candidate gene ACS1 (Prupe.6G214400; old nomenclature: ppa004987m). Scaffold 7 (G7) was the shortest at only 19 SNPs and four INDELS. However, the candidate gene CTR1, associated with the ethylene-responsive protein kinase Le-CTR1 (Prupe.7G117700; old nomenclature: ppa001532m) was placed in the middle of this scaffold. Although G8 was associated with the second-largest chromosome, with a total of 56 SNPs and 18 INDELS, no candidate genes associated with the differential phenotypic classes were found.

### Phylogenetic analysis of ethylene and sorbitol genes

**Figure 3:**
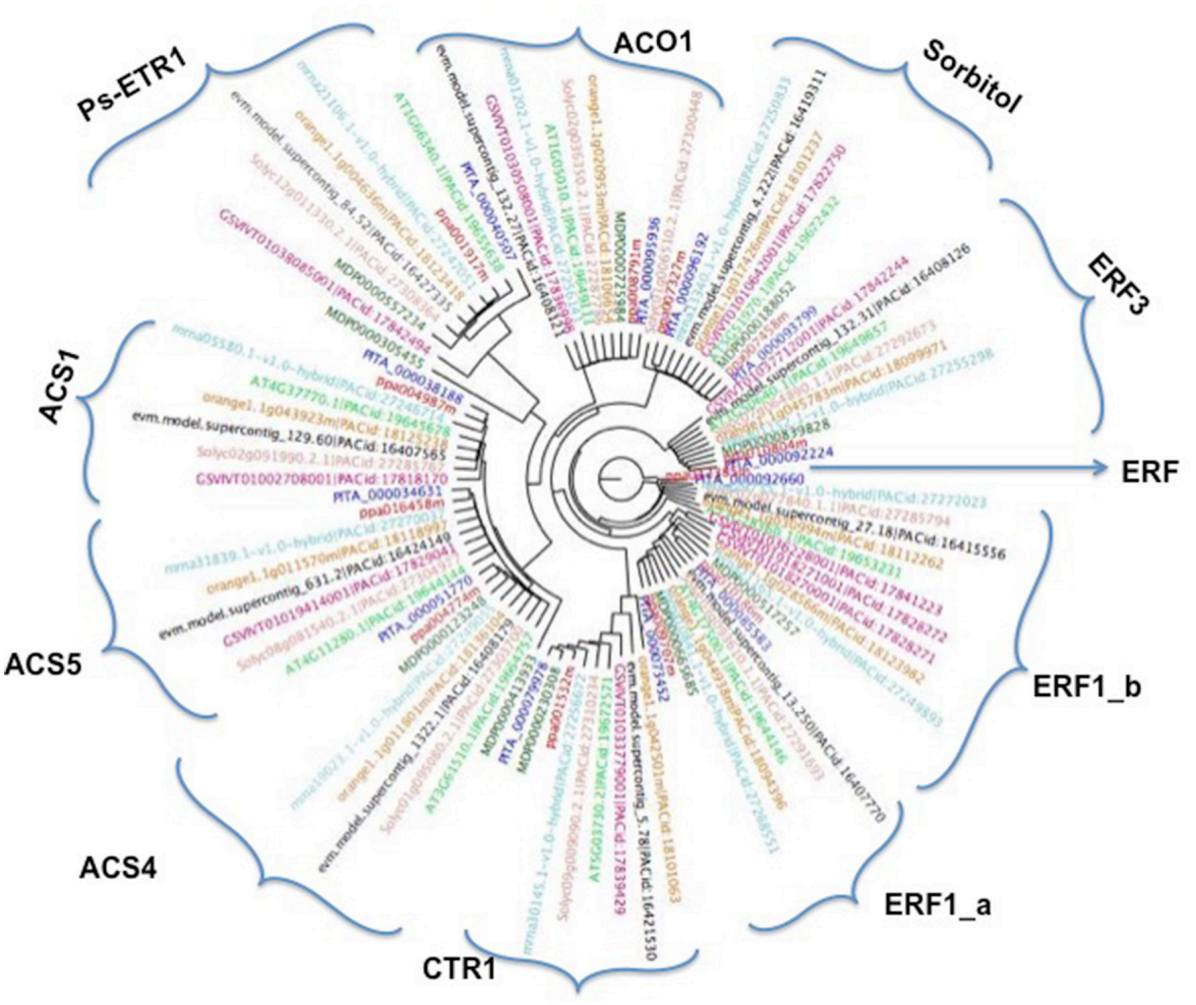
Phylogenetic tree by using the maximum-likelihood approach (RAxML) of the 10 ethylene and two sorbitol genes and their orthologs in *Malus domestica* (MD), *Arabidopsis thaliana* (AT), *Citrus clementina* (orange), *Fragaria vesca* (mrna), *Solanum lycopersicum* (Solyc), *Vitis vinifera* (GSV), *Carica papaya* (evm) *Prunus persica* (ppa) and *Pinus taeda* (PITA).

To study the evolutionary relationships between ethylene and sorbitol genes from peach, other species such as *Arabidopsis*, poplar, citrus, strawberry, pine, apple, papaya, tomato, and grape were used within this study. A phylogenetic tree was created based on the alignment of their amino acid sequences. This study was carried out using genome assemblies obtained from Phytozome. The maximum-likelihood (ML) phylogenetic tree allowed us to estimate the evolutionary relationships among the sequences (Figure 3). The two sorbitol dehydrogenase genes clearly fell into a distinct clade, indicating that this natural group is well-defined and presents high sequence identity among its members. In contrast, the ethylene group, which includes ACO1, ACS1, ACS4, ACS5, CTR1, ERF1, ERF3, and ethylene-receptor genes, was widely distributed and clustered into 10 different groups. The topology of the ML phylogenetic tree, showed ethylene genes clustering according to their similar biochemical activity, provided a framework for understanding the ethylene pathway in plum, suggesting that in plum and the other plant species, ethylene orthologs may play similar roles in ripening regulation. ACS1, ACS4, and ACS5 clustered in a major cluster together, although each gene was divided into different groups with their respective orthologs. The other ACO1 gene did not cluster in the same branches as its relatives. This may be due to annotation of this protein in peach as a member of the 2OG-Fe (II) oxigenase superfamily, while the other proteins ACS1, ACS4 and ACS5 were annotated as an aminotransferase class I and II, suggesting that they play different roles during ripening in plants. The other proteins with an AP2 domain, the ERF series, also clustered very close to each other, although each group formed its own branch. The other two groups, CTR1 and ETR1, clustered in different independent clades, but within the major branch of all the ethylene proteins and far from the two sorbitol genes.

## Discussion

In this work, we used whole-genome sequencing to identify genotypes associated with different fruit softening patterns in six Japanese plum cultivars derived from “Santa Rosa” with different climacteric responses. The “Santa Rosa” mutant series, described for the first time in the Rosacea family, exhibited three distinct ripening patterns: climacteric, suppressed-climacteric and non-climacteric (Minas et al., 2015). Most plum fruits, particularly the cultivar “Santa Rosa,” are climacteric but its bud-sport mutant “Sweet Miriam” is a non-climacteric (Minas et al., 2015). Although a previous molecular analysis using microsatellite markers showed the same genetic background, the existence of two different ripening behaviors in the fruits has led us to further investigate the possible differences among key ethylene and sorbitol gene families. The limited diversity study with 10 SSR markers to determine the genetic relationship of a group of climacteric, suppressed-climacteric and non-climacteric cultivars failed to distinguish the six cultivars belonging to the three different phenotypic and ripening patters (Minas et al., 2015). These cultivars originated as somatic mutations of the climacteric “Santa Rosa” and the likely low number of mutations in their genomes would not easily be detectable, using a set of 10 SSR markers.

Minor and structural variants are a common form of genome natural diversity that represents different types of genomic modifications. These have almost never been studied in plants and may have broad implications for model organism research, evolutionary biology and crop sciences. In plant breeding, the variants most widely studied are SNPs, since they are more efficiently manipulated and because minor changes may code for a single amino acid that may result in a functional change in the coded protein. The study of DNA sequence variation has been transformed by recent advances in DNA sequencing technology. Determining the functional consequences of sequence variant alleles offers potential insight on how genotype influences phenotype. Even within protein coding regions of the genome, establishing the consequences of variation on gene and protein function is challenging and requires substantial laboratory investigation. The recent introduction of next generation sequencing (NGS) technologies represents a major revolution in providing new tools to identify the genes and/or genomic intervals controlling important traits for selection in breeding programs. In perennial fruit trees with long generation times and large adult plants, the impact of these techniques is greater. Dissection of complex traits in many important tree species has become possible through the availability of genome sequences obtained by high-throughput DNA sequencing technologies, combined with phenotypic variation data. Such techniques offer shortcuts to discover candidate genes linked to selected traits and simplify analysis of diversity in a population.

In this study, 35,431 SNPs and 6,149 INDELS were initially identified among the six Japanese plum cultivars that had a high variability of representation along scaffolds of the peach genome. The high rate of variation observed in scaffold 1 may be because of the presence of more recombination hotspots, as previously reported (Salazar et al., 2017). Using SNP markers in an F1 Japanese plum population, Salazar et al. (2017) reported LG5 as the shortest group; however, in our population, scaffold 7 had the fewest markers, which is in agreement with a new Japanese plum “Angeleno x Aurora” genetic map (Carrasco and Silva, personal communication, August 2017).

The increasing availability of whole-genome sequencing provides a new opportunity to investigate genetic variation for ripening genes in plum, since the variation in gene copy numbers among plant genomes is understudied and poorly characterized. Unlike variation involving single-nucleotide changes, data on variation in copy number is difficult to collect and few tools exist for analyzing variation between individuals.

Twelve potential candidate genes with significant CNV were associated with ethylene perception and signal transduction components: ERT, CRT, the ACC-synthase gene family (ACS), the ethylene-responsive transcriptional factor (ERF), and two sorbitol dehydrogenase genes. Out of 12 targeted plum genes, Ps-ACO1, Ps-ACS1, Ps-ACS4, Ps-ACS1, Ps-CTR1, Ps-ERF1, Ps-ERF1a, Ps-ERF1b, Ps-ETR1, Ps-ERF3, Ps-Sorbitol1 and Ps-Sorbitol2, only Ps-ACS3 showed no differences in CNV among the examined cultivars. Among the aforementioned 11 genes, seven genes had SNPs with a gene effect of either synonymous variant or 5′prime UTR premature start codon gain variant. SNPs in upstream and downstream regions cause phenotypic variations when they activate or suppress gene expression by causing substitutions in regulatory sequences. This may have occurred in the non-climacteric and suppressed-climacteric type cultivars, where a loss-of-function mutation could cause drastic alteration in fruit ripening and sorbitol phenotypes. The other genomic variants identified as potential candidate genes were five different INDELS. Prupe.3G209900 had a substitution of TGGTACAC by T and Prupe.5G061800 had a change in gene sequences from T to a TATATATAA. The other three INDELS had only two-nucleotide substitutions.

CNV are likely to have significant functional impacts on genes and may explain additional phenotypic variation not captured by SNPs (Manolio et al., 2009). When CNV change the number of copies of a given gene, they alter its level of expression, leading to genetic and phenotypic difference between individuals and populations. Several studies in plants found that genes affected by CNVs are associated with important agronomic traits. In *Oryza sativa*, a CNV at Grain Length locus on chromosome 7 contributed to grain size diversity (Wang et al., 2012). CNVs at the Rhg1 locus mediate resistance to soybean cyst nematode (Cook et al., 2012). In barley, increased copy number of a boron transporter gene (Bot1) conferred tolerance to boron toxicity (Sutton et al., 2007). However, exploration of the extent and role of CNVs in plants is just beginning. The study of genomic regions that contain gene copies and structural variation is a major challenge in modern genomics. The variation we observed in the 11 genes may have functional consequences related to sorbitol synthesis, perception and metabolism, respectively, and as a consequence be the key elements responsible to the three phenotypic and ripening patterns found in the six plum cultivars.

Based on the ML tree topology, ethylene and sorbitol genes were classified in 11 subfamilies grouped according to their sequence similarities with other plant species. The subfamilies were supported not only by phylogenetic analysis, but also by gene structure. All families had clearly defined gene clusters for the same biochemical functions. The two sorbitol genes grouped together while the 10 ethylene genes clustered in independent clades with their orthologs from the other plant species. This suggests that the genes evolved from a common ancestor before the divergence of specific lineages. Thus, based on the distribution of the clades, we may suggest that gene function diversification for the ripening pathway happened prior to family extension. Therefore, most ripening genes may have been established early in a plant evolution, before the divergence of plant lineages. This early origin of most subfamilies suggests that the genes have central roles in regulating common ripening pathways of different plant lineages, implying that this family of genes has expanded over the course of plant evolution.

Additionally, the non-synonymous versus synonymous mutation rate (*D*_*n*_/*D*_*s*_) among the ethylene and sorbitol candidate gene pairs were very similar in the relationships observed in the sequences of the genes. These observations imply that a greater proportion of non-synonymous than synonymous variants were relatively rare as the result of ongoing purifying selection. In our study, most estimated *D*_*n*_/*D*_*s*_ values were <1, meaning that the duplicated ethylene and sorbitol sequences were under purifying selection pressure (Table 4). The study of the forces of mutation and selection and their effect at the molecular level is crucial to understand how species have evolved over time (Lynch, 2010) and how positive selection accelerated change over evolutionary time. We found a greater proportion of sites with negative selection coefficients. However, one duplicate gene pair, Prupe.3G209900/Prupe.5G106200, underwent positive selection after being separated by duplication, implying that functional divergence of the duplicated genes may have been accelerated by positive selection over evolutionary time.

**Table 4:** Mutation rate of candidate genes associated with ethylene and sorbitol biosynthesis.

**Paralogous pairs**		**ds**	**dn**	**ds/dn**
Prupe.2G176900	Prupe.2G288800	2.6552	3.2352	0.8207
Prupe.3G209900	Prupe.5G106200	3.7189	3.3032	1.1259
Prupe.3G209900	Prupe.3G209100	2.1524	2.2982	0.9366
Prupe.2G288800	Prupe.6G214400	2.3015	2.3671	0.9723
Prupe.7G117700	Prupe.6G214400	2.852	3.4697	0.822
Prupe.5G106200	Prupe.4G051200	2.3744	3.1643	0.7504
Prupe.3G209100	Prupe.6G214400	2.2955	3.0286	0.758
Prupe.6G214400	Prupe.6G214400	3.0832	3.847	0.8015
Prupe.4G240300	Prupe.5G106200	2.9239	3.3693	0.8678
Prupe.4G051200	Prupe.5G061800	1.7203	2.0727	0.83
Prupe.1G556000	Prupe.2G272300	1.9929	2.7415	0.7269
Prupe.2G272300	Prupe.6G214400	3.2226	3.4072	0.9458
Prupe.5G106200	Prupe.6G214400	2.4745	3.2951	0.7509

## Conclusion

This series of Japanese plum mutants proved ideal for study of the fruit ripening syndrome in *Prunus* species. Twelve candidate genes associated with ethylene and sorbitol biosynthesis between the two main phenotypes (climacteric vs. non-climacteric) present a good starting point for further studies into mRNA accumulation patterns during on-tree fruit development and maturation. These genes seemed to affect regulation of ACC synthase, ethylene receptors, constitutive triple response, and sorbitol dehydrogenase genes. We successfully employed whole genome sequencing to detect genomic variants associated with different ripening patterns of a reference plum cultivar Santa Rosa and five somatic mutants. To develop resources for Japanese plum genetic studies, we created a map with all the filtered SNPs/INDELS. This provides a valuable resource for future association studies such as GWAS or QTL analysis to relate phenotypes to genotypes. On the other hand, we studied the evolutionary relationships between ethylene and sorbitol genes in several model plant species, concluding that these family genes have evolved faster and prior to the divergence of plant lineages. Finally, the physical map with the locations of the SNP/INDELS, that differed among the cultivars and were associated with genes involved in ethylene biosynthesis, provides useful reference to the plum breeding community and has a potential in aiding practical orchard manipulations. Thus, this “Santa Rosa” plum series provides a novel fruit system that can be exploited in order to develop markers that may assist breeders in providing high-quality plum cultivars with extensive market life.

## Author contributions

AFM, CS, GM, KG, and CC: designed the experiments; AFM and CS: analyzed the data; AFM: wrote the manuscript; All authors read and approved the final manuscript.

### Conflict of interest statement

The authors declare that the research was conducted in the absence of any commercial or financial relationships that could be construed as a potential conflict of interest.

## Supplementary material

Supplementary Figure 1 Total number of variant sites identified after filtering for depth, allele frequency, mapping quality, and coding region.

Supplementary Figure 2 **(A)** A neighbor joining phylogenetic tree from SNPs among the six cultivars. **(B)** A PCoA plot depicting the dimensional relationships among the six cultivars using SNPs.

Supplementary Table 1 Sequencing summary for plum cultivars.

Supplementary Table 2 Summary of the unique variants effects (SNP/INDEL) for each of the plum cultivars.

Supplementary Table 3 Discriminatory SNP variants among the 6 plum cultivars where at least one cultivar differs.

Supplementary Table 4 Discriminatory INDEL variants among the 6 plum cultivars where at least one cultivar differs.

Supplementary Table 5 Summary of the copy number variations when comparing **(a)** Santa Rosa vs. Sweet Mariam, **(b)** Santa Rosa vs. Late Santa Rosa, Casselman and Roysum and **(c)** Sweet Mariam vs. Late Santa Rosa, Casselman, and Roysum.

Supplementary Table 6 This table shows the 281 SNP/INDELS differences with their respectives chromosome location between Santa Rosa and the rest of plum cultivars after filtering and based of gene function of the SNP.

Supplementary Table 7 This table shows the 108 SNP/INDELS differences between Santa Rosa and Sweet Mariam after filtering and based of gene function of the SNP.

Supplementary Table 8 This table shows the 260 SNP/INDELS differences between Sweet Mariam and the suppressed cultivars, which are July Santa Rosa, Late Santa Rosa, Casselman, and Roysum after filtering and based of gene function of the SNP.

Supplementary Table 9 This table shows the 204 SNP/INDELS diferences between climateric (July Santa Rosa, Santa Rosa and Late Santa Rosa) vs. the non-climateric Sweet Mariam after filtering and based of gene function of the SNP.
