# Supplementary figures and images for: Genomic Sequencing of Japanese Plum (Prunus salicina Lindl.) Mutants Provides a New Model for Rosaceae Fruit Ripening Studies
Source: Front Plant Sci. 2018 Feb 19;9:21. doi: 10.3389/fpls.2018.00021 (PMC5825990; doi:10.3389/fpls.2018.00021)

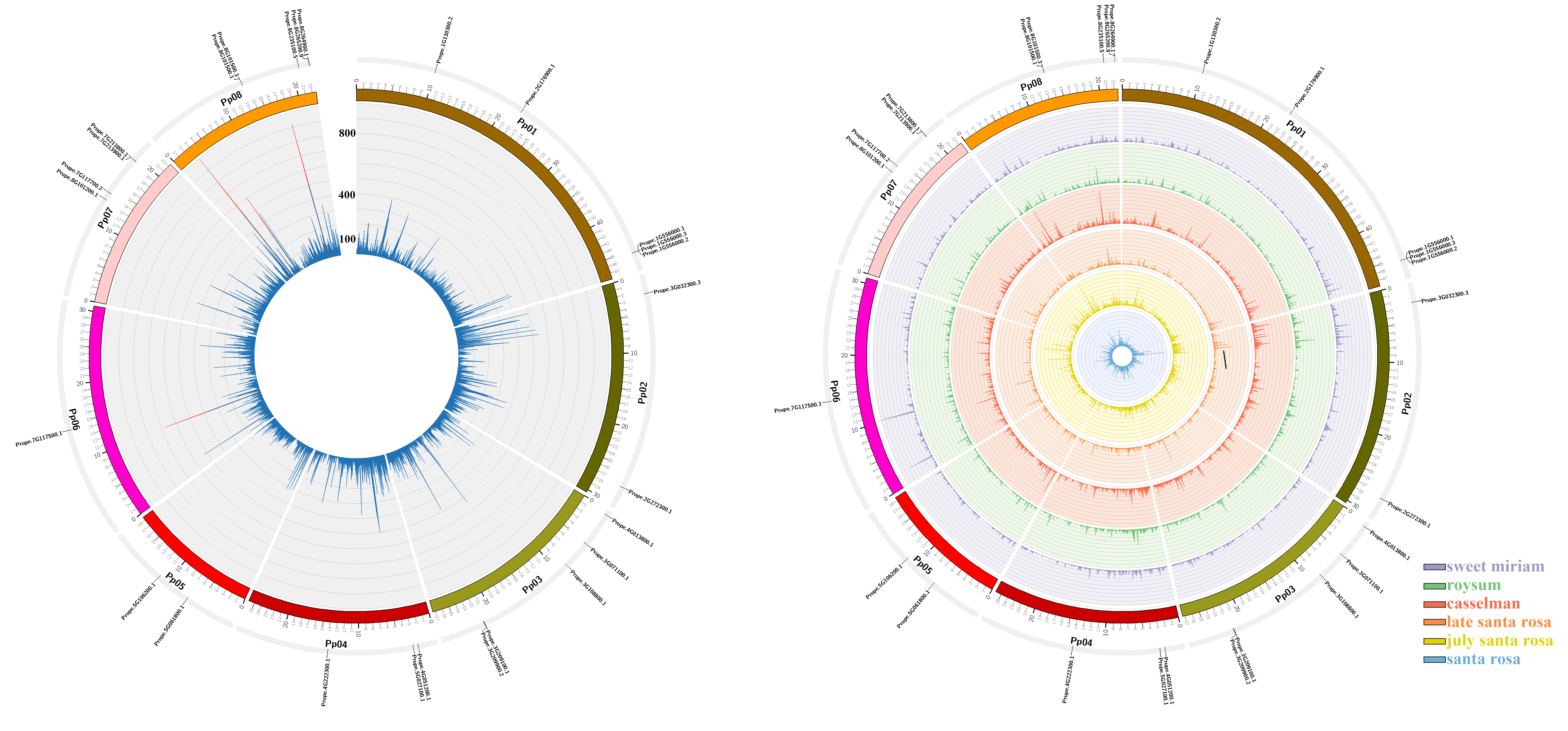

Supplement: Supplementary Figure 1 — Total number of variant sites identified after filtering for depth, allele frequency, mapping quality, and coding region. [file Image1.PNG]

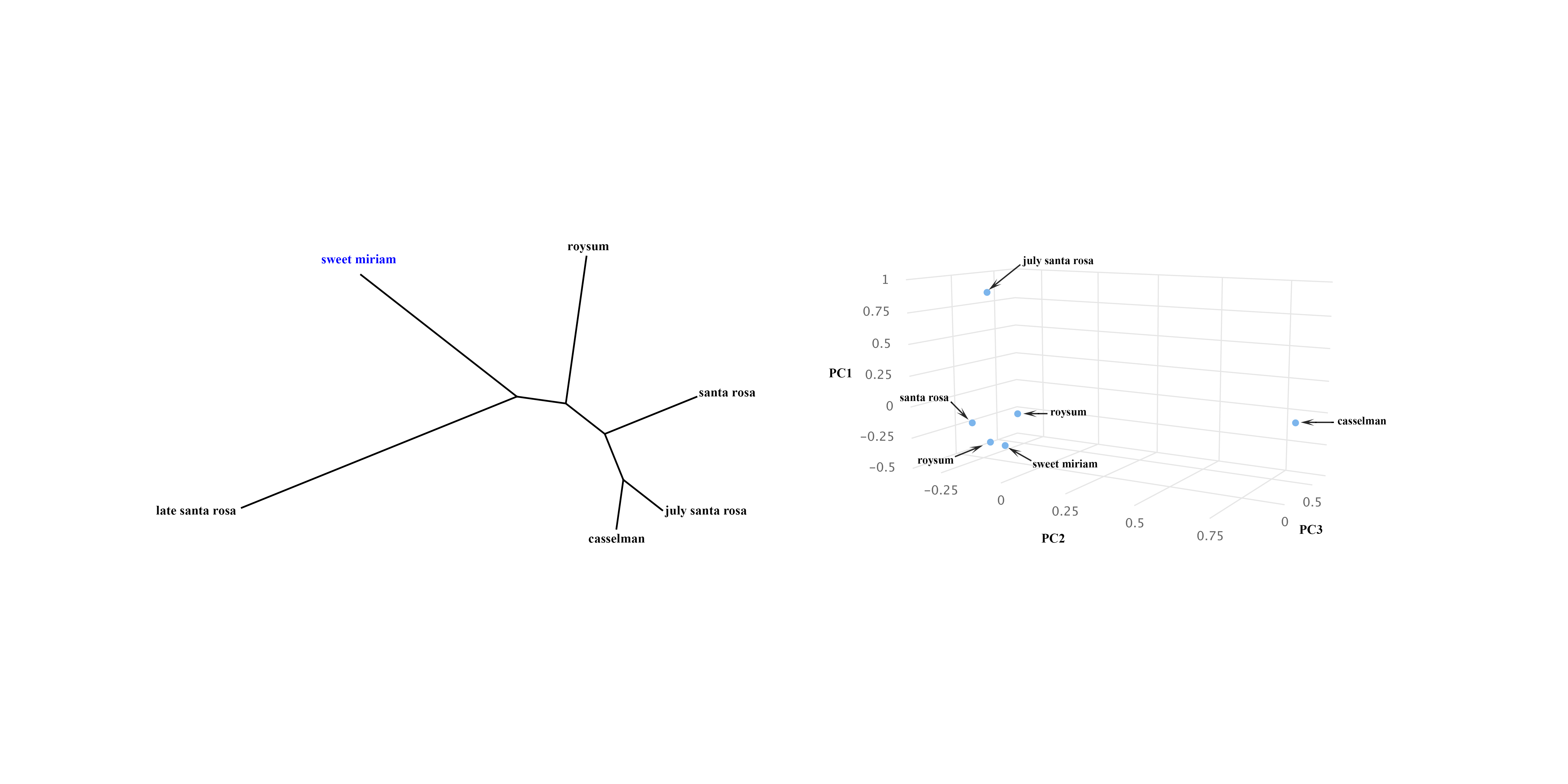

Supplement: Supplementary Figure 2 — (A) A neighbor joining phylogenetic tree from SNPs among the six cultivars. (B) A PCoA plot depicting the dimensional relationships among the six cultivars using SNPs. [file Image2.PNG]
